# Efflux pumps of Gram-negative bacteria: what they do, how they do it, with what and how to deal with them

**DOI:** 10.3389/fphar.2013.00168

**Published:** 2014-01-03

**Authors:** Leonard Amaral, Ana Martins, Gabriella Spengler, Joseph Molnar

**Affiliations:** ^1^Travel Medicine of the Centro de Malária e Doenças Tropicais, Institute of Hygiene and Tropical Medicine, Universidade Nova de LisboaLisbon, Portugal; ^2^Institute of Medical Microbiology and Immunobiology, University of SzegedSzeged, Hungary; ^3^Unit of Parasitology and Medical Microbiology, Institute of Hygiene and Tropical Medicine, Universidade Nova de LisboaLisbon, Portugal

**Keywords:** Gram-negative bacteria, inhibition, efflux pumps, quorum sensing, biofilms, genetic responses to antibiotic exposure, inhibitory agents of efflux pumps and QS, methods for identification of bacteria that over-express efflux pumps

## Abstract

This review discusses the relationship of the efflux pump (EP) system of Gram-negative bacteria to other antibiotic resistance mechanisms of the bacterium such as quorum sensing, biofilms, two component regulons, etc. The genetic responses of a Gram-negative to an antibiotic that render it immune to an antibiotic are also discussed. Lastly, the methods that have been developed for the identification of bacteria that over-express their EP system are presented in detail. Phenothiazines are well-known antipsychotic drugs with reported activity against bacterial EPs and other ancillary antibiotic mechanisms of the organism. Therefore these compounds will also be discussed.

## INTRODUCTION

Bacterial efflux pumps (EPs) are proteins that are localized and imbedded in the plasma membrane of the bacterium and whose function is to recognize noxious agents that have penetrated the protective cell wall of the organism and reached the periplasm or cytoplasm, and extrude them before they reach their intended targets (Amaral et al., 2008, 2010b, 2011b; Pagès and Amaral, 2009; Pagès et al., 2011). Moreover, EPs also recognize toxic compounds that are products of metabolism of the bacterium and hence perform excretory functions as well (Li and Nikaido, 2009; Nikaido, 2011). In other words, EPs are transporters of noxious compounds from within the bacterial cell to the external environment. With the possible exception of excretory functions, EPs utilize sources of energy for their function inasmuch as they transport compounds against a concentration gradient. There are two particularly distinguished types of immediate sources of energy utilized by the different known families of EPs: ATP (Marshall and Piddock, 1997; Lewis, 2001; Lorca et al., 2007; Moitra et al., 2011) and the proton motive force (PMF; Amaral et al., 2008, 2010b, 2011b; Li and Nikaido, 2009; Pagès and Amaral, 2009; Nikaido, 2011; Pagès et al., 2011; Spengler et al., 2012). For example, ABC transporters directly utilize ATP for their energy source. These ABC transporter proteins consist of two domains; one that is embedded in the plasma membrane and other is on the medial side of the plasma membrane. The domain that is on the medial side of the plasma membrane has two sites for the binding of the substrate and two sites for the binding and hydrolysis of ATP. Subsequent to the recognition of the noxious agent and its binding to the ABC transporter, ATP is hydrolyzed providing the needed energy for the conformational changes of the transporter that promote the extrusion of the noxious agent to the environment (Marshall and Piddock, 1997; Bhattacharjee et al., 2000; Lewis, 2001; Lorca et al., 2007; Moitra et al., 2011). The precise structural changes that take place as well as the means by which the transporter recognizes structurally unrelated compounds is not yet completely understood. Unlike the ABC transporters, members of resistance nodulation division (RND) family obtain their energy from the PMF established as the result of cellular metabolism. Protons that are not used for coupling with molecular oxygen are exported to the surface of the cell (Mulkidjanian et al., 2005, 2006; Mulkidjanian, 2006) where they are distributed and bound to components of the protective lipopolysaccharide (LPS) layer and basic amino acids of the outer cell wall of Gram-negative (Roberts, 1996) and Gram-positive bacteria (Nikaido, 2003). The differential distribution of hydronium ions relative to their concentration in the milieu results in a pH at the surface of the cell that is two to three pH units lower than that of the milieu (Mulkidjanian et al., 2005, 2006; Mulkidjanian, 2006). These surface bound hydronium ions can travel through porins (Achouak et al., 2001; Pagès et al., 2008) that carry water into the periplasm and hence they contribute to the concentration of hydronium ions at the periplasmic surface of the plasma membrane. Because the concentration of hydronium ions is greater at the periplasm than that at the plasma membrane surface medial to the cytoplasm, an electrochemical gradient results: the PMF (Prebble, 1996). These hydronium ions can therefore move in accordance to the established gradient from the periplasm to the cytoplasmic medial side of the plasma membrane via porins on the plasma membrane. Before the movement of hydronium ions is further discussed as the source of energy from the PMF, the structure and mechanism of the main EP of *Escherichia coli* (which belongs to the RND family) need addressing.

**FIGURE 1:**
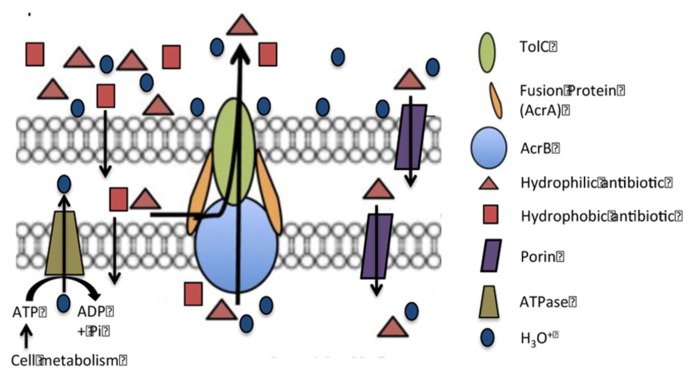
**Model of the AcrAB-TolC efflux pump of a Gram-negative bacterium.** Hypothesis. At near neutral pH, hydronium ions from hydrolysis of ATP by ATP synthase pass through the AcrB transporter, reduce the pH to a point that causes the release of the substrate. When the hydronium ions reach the surface of the cell they are distributed over that surface and bind to lipopolysaccharides and basic amino acids. When there is a need for hydronium ions for activity of the efflux pump, the pH is lower than neutral and the hydrolysis of ATP is not favored, hydronium ions from the surface of cell due to the PMF move through the porins and reach the transporter where they are pushed through the transporter by the peristaltic action caused by the fusion proteins. Substrates bound to the transporter dissociate when the pH is reduced by the flow of hydronium ions and are carried out by the flow of water.

The main EP of *E. coli* is the AcrAB-TolC efflux pump (Ma et al., 1995; Okusu et al., 1996; Viveiros et al., 2005). In situations when this pump is deleted or deactivated, its function is replaced by another RND efflux pump the AcrEF-TolC pump (Viveiros et al., 2005). Both EPs consist of three distinct proteins. The transporter component of the AcrAB-TolC pump, AcrB, is attached to the plasma membrane and coded by the gene *acrB*. There are two fusion proteins, AcrA, coded by the gene *acrA*, that flank the AcrB transporter and are believed to assist the movement of a substrate through the AcrB transporter by peristaltic action driving water through the transporter (Nikaido, 2011). The third component of the AcrAB-TolC pump is the TolC channel which is contiguous with the AcrB transporter and provides a conduit for the extrusion of the substrate (Lorca et al., 2007). The TolC protein is also part of other tri-unit EPs of the organism (Nikaido, 2011). Even though the AcrAB-TolC efflux pump has been studied for three decades and has been shown to extrude a large variety of unrelated compounds with widely different structures (Nikaido, 2011), its structure in the plasma membrane has not yet been completely defined. Nevertheless, the means for the recognition of the substrate appear to involve a pocket within the transporter and defined by a phenylalanine residue (Eicher et al., 2012). Studies employing fluorochromes recognized by the AcrB transporter indicate that the binding and release of the substrate are pH dependent (Su and Yu, 2007). At low pH the dissociation of the substrate is high and at high pH it is very slow. Therefore, in a physiological environment of pH 7, one would expect that the pump would be very ineffective since the dissociation of the substrate would be slow or none at all. How then does the pump continue to function if the release of the substrate is limited? The function of the pump at environmental conditions (*ca.* pH 7) must involve conditions, which decrease the pH of the internal cavity of the pump to which the substrate is bound and therefore afford the extrusion of the substrate possible. To accomplish this at physiological pH, we have postulated that the decrease in pH within the pocket takes place by the generation of hydronium ions from metabolism (Amaral et al., 2011b), which pass from the cytoplasmic side of the plasma membrane through the transporter. At lower pH (below *ca.* 6.5), hydronium ions can be diverted by the PMF from the surface of the cell to the periplasm via porins and then from the periplasm to the medial side of the plasma membrane via another porin. Because the transporter can “vacuum” the substrate from either the periplasmic or cytoplasmic medial sides of the plasma membrane, hydronium ions must also gain access to the internal component of the pump thereby affording the needed drop of pH for release of the substrate and subsequent extrusion via the TolC channel. The differential pH function of the F_0_–F_1_ ATP synthase insures that hydronium ions are generated from the hydrolysis of ATP at high pH or are used for the synthesis of ATP at low pH (Walker et al., 1984; Feniouk and Junge, 2005; Padan et al., 2005). The model proposed by **Figure 1** describes the mechanism for the function of the RND AcrAB-TolC efflux pump of Gram-negatives.

## INDUCING GENETIC UP-REGULATION OF EFFLUX PUMP BY AN ANTIBIOTIC AND DOWN-REGULATION OF PORINS

Apart from chromosomal mutation or acquisition of plasmids or mobile genetic elements encoding resistance determinants, a Gram-negative bacterium can increase its antibiotic resistance by preventing the antibiotic from entering the cell. This can be achieved by the control of the outer membrane permeability (decreasing the number of porins that allow the compounds to enter the cell) and/or by the increasing the effectiveness of the efflux (active pumping out) of antibiotics, usually increasing the number of pumps available (Nikaido, 2001; Gootz, 2006; Piddock, 2006). The effectiveness of the outer membrane of Gram-negative bacteria as a barrier, in most cases, only delays the influx of various antibiotics, detergents, and dyes. Intrinsic resistance to antibiotic agents is mainly due to EPs enabling bacterium to survive in the presence of these noxious agents (Nikaido, 2001; Davin-Regli and Pagès, 2006). If the concentration of the noxious agent exceeds the capacity of the intrinsic EP to extrude the agent, the over-expression of the main EP takes place, resulting in a multidrug resistant (MDR) phenotype (Nikaido, 2001; Gootz, 2006; Piddock, 2006). MDR is now known to be a prevalent form of clinical resistance (Nikaido, 2001).

Under laboratory conditions, induction of high-level resistance to tetracycline (TET) in *E. coli* K-12 strain results in the over-expression of nine major inner membrane transporter genes, with the *acrB* being the most expressed (Basle et al., 2006). The cited studies demonstrate a clear connection between the induced activity of the AcrAB system and TET induced resistance. Moreover, resistance can be reversed by either transfer to drug free medium, by the use of Phe-Arg-β-naphthylamide (PAβN), an inhibitor of the AcrAB efflux pump system or by the phenothiazine thioridazine (TZ; Cowan et al., 1992; Nikaido, 2003; Basle et al., 2006). Besides becoming resistant to TET, the induced strain becomes resistant to a variety of other antibiotics, detergents, and dyes that are not substrates of the AcrAB system (Nikaido, 2001; Davin-Regli and Pagès, 2006; Gootz, 2006; Piddock, 2006).

As previously mentioned, in *E. coli*, permeability of the outer membrane is also controlled by the regulation of the expression of porins (Pages et al., 2005). The two major outer membrane proteins (OMPs) in *E. coli* are OmpC and OmpF, consist of three 16-stranded β-barrels defining a trans-membrane pore in the outer membrane porin (Cowan et al., 1992; Nikaido, 2003). These OMPs are highly expressed under optimal environmental conditions, their level of expression is adjusted when it is necessary to minimize penetration of noxious compounds or maximize access to nutrients (Liu and Ferenci, 2001; Basle et al., 2006). For example, the level of expression of the porins OmpC and OmpF not only controls the permeability of the outer membrane to glucose and nitrogen uptake under nutrient limitation (Ferenci, 2005; Castillo-Keller et al., 2006), but may also be differentially regulated by the concentration of certain antibiotics in the environment (Randall and Woodward, 2002; Castillo-Keller et al., 2006). Immunoblot and antibody recognition analysis has confirmed that OmpF and OmpC synthesis is reduced or markedly reduced, respectively, during the adaptation process and accompany the up-regulation of EPs observed during the process of exposure to TET (Viveiros et al., 2007). These results are consistent with the notion that when the bacterium is placed under antibiotic stress, under conditions that permit it to adjust (namely slow exposure to sub-lethal concentrations of the antibiotic and nutrient availability), antibiotic resistance is increased by the up-regulation of EPs and down-regulation of porins (Delihas and Forst, 2001; Chen et al., 2004).

Expression of the genes coding for OmpC and OmpF is regulated by a two-component signal transduction regulatory system consisting of the OmpR and EnvZ proteins (Hall and Silhavy, 1981). Moreover, over-expression of OmpX, a porin structurally related to the eight-β strand OmpA (a major OMP involved in the stabilization of the bacterial membrane), leads to a decrease in the expression of OmpC and OmpF porins and a decreased susceptibility to β-lactams and other antibiotics in *E. coli* (Dupont et al., 2004). However, mutants with decreased expression of porins show only small increases in the minimum inhibitory concentration (MIC) of relevant antibiotics, suggesting that the complete shut-down of influx of small molecules into *E. coli* does not readily occur (Ma et al., 1994).

The sequential expression of genes that are involved in a response to TET are depicted by **Table 1** and are discussed in terms of relationships that have been established for regulatory and responding genes.

**Table 1:** Relative expression of *E. coli* genes after adaptation to increasing concentration of tetracycline (Viveiros et al., 2007).

@ Tet MIC (μg/ml)	Relative expression of *E. coli *genes																		
	*soxS*	*rob*	*marA*	*marB*	*marR*	*acrA*	*acrB*	*acrE*	*acrF*	*emrD*	*mdfA*	*yhiV*	*TolC*
**1.5**	2.8	0.5	3.0	0.5	**2.0**	1.8	1.8	1.2	1.5	1.5	1.5	**2.2**	1.5
**4.0**	**3.5**	**2.8**	**5.5**	**4.5**	**4.3**	**6.0**	**5.0**	**2.4**	**2.8**	**4.9**	**3.8**	**5.8**	**5.8**
**10.0**	**2.2**	**1.7**	**9.7**	**4,4**	**3.1**	**12.8**	**9.4**	**2.4**	**2.6**	1.7	**3.7**	**6.4**	**4.5**

*Relative expression of efflux pump regulators and efflux pump transporter genes at each level of induced resistance to tetracycline. Data from three independent total mRNA extractions of *E. coli* AG100 physiologically adapted to increasing concentrations of TET compared to its parental non-induced strain grown in the absence of TET as described in Viveiros et al. (2007). A ratio of 1 corresponds to no alteration in expression compared with untreated control cells. Ratios above 2.0 are considered significant. Values were corrected for standard deviation range. Bold expressions are the major genes responding to serial exposure to increasing concentrations of tetracycline each of which is identified as the MIC of that exposure step. Note that *marA, acrA, acrB*, and *yhiV* continue to express progressive increase of activity noted at the end of each MIC step of exposure to an increase of TET concentration. The activity of other genes such as *ompF*, etc. are not shown in ***Table 1*** but are discussed in the text.
*

OmpA is considered to be a structural OMP that contributes to the integrity of the cell envelope as a tri-barrel structure (Viveiros et al., 2007). It does not appear to have a role in functions normally attributed to porins. However, in the middle of the adaptation process a transient increase of *ompA* expression is noted, which could mean a need for structural strengthening of the cell envelope while protein synthesis is reduced due to the exposure of increasing concentrations of TET. However, when the bacterial cells are completely adapted to high concentrations of TET, this high concentration of TET should result in TET reaching binding sites of the 30S ribosomal subunits and therefore, affect the synthesis of many proteins. However, if this were to be the case, the increase of the AcrB and AcrA proteins should also be affected, and this was not the case as shown by the cited studies (Viveiros et al., 2007). The same authors also suggested that the down-regulation of porins C and F takes place by the increased synthesis of proteases, which degrade the *de novo* porins C and F (Viveiros et al., 2007).

Porin A which contributes to the stability of the outer membrane is not affected by the proteases and consequently the integrity of the outer membrane is maintained, thereby complementing the resistance afforded by down regulation of porins C and F.

The ompX gene codes for the outer membrane protein OmpX and over-production of this protein induces a reduction of the porin level in *Enterobacter aerogenes* (Dupont et al., 2004). In *E. coli*, during adaptation to TET, *ompX* activity is the highest of all of the genes evaluated. Therefore, the regulatory role for this OMP appears to involve a direct effect on porin assembly.

## THE STRESS GENES *soxS* AND *rob*

During the adaptation process of *E. coli* K12 to step-wise increasing concentrations of TET, expression of the stress-response gene *soxS* can be increased up to 3.5 times comparing to the untreated control (Viveiros et al., 2007). However, when the strain is adapted to high concentrations of TET, response of the gene is reduced to a level below that initially observed, suggesting that the stress gene *soxS* performs its functions quite early under conditions of antibiotic pressure (Viveiros et al., 2007). The other stress-response gene, *rob*, responds later; however, as in the case of the other stress-response gene *soxS*, the increased activity is apparently not required for higher levels of resistance (i.e., 10 mg/L) (Viveiros et al., 2007). The gene *rob* has been reported to respond to exposure to solvents, detergents, metals (Ariza et al., 1995; Nakajima et al., 1995), and antibiotics (Viveiros et al., 2007).

## REGULATORY GENES *marA, marB*, AND *marR*

The regulatory product of gene *marR* is known to down-regulate the activity of genes *marA* and *marB* by binding to the promoter region of the operator *marO*. Because TET is known to bind to the product of *marR*, and this produces an MDR phenotype (Pratt and Silhavy, 1996), once the repressor activity is inhibited, the universal regulator *marA* would be expected to increase its activity. Indeed, of all regulator genes, it is *marA* that is increased to the highest level (9.7-fold) at the time when the organism has developed resistance to TET (10 mg/L). Although nothing is known about the role of *marB* or the relationship between *marA* and *marB* during MDR phenotypic expression, the cited study suggested that *marB* might precede *marA*, during the development of TET resistance (Viveiros et al., 2007).

## ANCILLARY GENES *micF, ompF* INVOLVED IN DEVELOPING RESISTANCE TO TETRACYCLINE

The genes *marA, rob*, and *soxS* regulate the activity of *micF* responsible by the down-regulation of OMPs (Guillier et al., 2006; Bohnert et al., 2007). In the former example of *E. coli* K12, the increase of activity of *micF* reaches its maximum level (comparable to that of *marA*) when the organism becomes resistant to 10 mg/L of TET. The over-production of MicF decreases the amount of OmpF mRNA (Bohnert et al., 2007); similarly MicC may have the same effect on OmpC mRNA stability.

The *ompR* and *envZ* genes are regulators of OMPs. The genes *ompR* and *envZ* belong to the two-component signaling family and modulate gene activities of *ompF* and *ompC*, the two major OMP genes that code for the tri-barrel porin. When *E. coli* is placed under stress, a cascade of gene activities is initiated, involving several global regulators such as MarA and MicF, which result in the down-regulation of porins. This down-regulation results in decreased activity of *ompF* and *ompC*. Whereas the increase in the expression of the *ompR* and *envZ* genes is maintained for the duration of exposure to increasing concentrations of TET, the response of the *ompA, ompC*, and *ompF* genes is transiently increased and subsequently reduced to levels comparable to those of the *E. coli* cells that were not exposed to TET. Because *micF* is considered to be a post-transcriptional regulator of porins, the activity of *ompF* and *ompC* may be related to the expression of this gene (Delihas and Forst, 2001). However, the porin mRNA level is similar to that produced in untreated cells. We may assume that, as previously reported (Delihas and Forst, 2001), the putative regulator of *ompX* becomes limited and cannot induce porin mRNA degradation.

## EFFLUX PUMP GENES *acrAB* AND THE OTHER EFFLUX PUMP TRANSPORTER GENES

The response of the genes coding to the AcrAB-TolC when the organism is initially exposed to increasing concentrations of TET below that of its MIC is marginal, suggesting that the normal activity of the operon is enough to respond to the pressure caused by sub-inhibitory concentrations of antibiotic. However, as the concentration of antibiotic (in the example: TET) increases, the expression of the genes coding to the AcrAB-TolC also increases as the organism becomes more and more resistant to the antibiotic. Moreover, the stress imposed during the early stages of exposure to TET requires the cooperation of all of the EPs, but their expression is reduced later on during the adaptation process, when both *acrB* and *yhiV* (a gene coding to an RND efflux pump of *E. coli* with significant homology to AcrB; Bohnert et al., 2007), reach their highest level of expression. The increased activity of the genes coding to the AcrAB-TolC is accompanied by the increased activity of the regulator *marA* and increased synthesis of AcrA.

## RELATIONSHIP OF THE MAIN EFFLUX PUMP SYSTEM TO THE ACCUMULATION OF MUTATIONS

As noted in the previous section, serial exposure to increasing concentrations of an antibiotic that is just below its MIC will promote progressive activation of genes that regulate and code for the components of the EP of the Gram-negative bacterium (Viveiros et al., 2005, 2007). However, if at any time during the above process, the highest concentration is maintained during further serial cultures, a number of further responses take place. Firstly, for a sequence of serial cultures, there is a corresponding increase in the expression of genes that regulate and code for the constituents of the main EP of the Gram-negative *E. coli* (Martins et al., 2009a, 2012). However, at a certain point, the expression of these genes begins to decline and simultaneously the organism begins to express an increasing number of mutations in key targets such as those that are present on the plasma membrane (example penicillin binding proteins), gyrase and in the 30S component of the ribosome (Martins et al., 2009a). Eventually, the expression of genes that regulate and code for the components of the EP reach basal levels of the wild-type (non-exposed bacterial strain). At this point, the protection of the bacterium from the presence of a constant concentration of an antibiotic comes from the accumulated mutations on key antibiotic targets (Martins et al., 2009a, 2012). Nevertheless, if the bacterium is transferred to antibiotic-free medium that contains its counterpart wild-type strain, within a few serial transfers, it ceases to exist. Therefore, whereas the accumulated mutations provide protection in a highly selective environment, that protection renders the organism less fit for survival (Martins et al., 2009a, 2012, 2013a). Similar approaches have yielded similar responses with mycobacteria (Martins et al., 2007; Viveiros et al., 2012) and staphylococci (Martins et al., 2011a) suggesting that the above responses to constant antibiotic stress are a common response of bacteria.

## SIMPLE METHODS FOR ASSESSMENT OF THE EFFLUX PUMP SYSTEM AND EVALUATION OF AGENTS FOR INHIBITORY ACTIVITY

Agents which reduce the activity of a given EP are referred in the literature as “efflux pump inhibitors” (EPIs) even though direct inhibition of the activity of the pump by binding of the agent is rarely shown or evident. Therefore some authors prefer to call them “efflux pump modulators.” Nevertheless, although there is now evidence that the reduced EP activity may be due other indirect pathways that decrease efflux, the term “EPI” will be retained in the discussion. Among the mechanisms by which EPIs act within the cell, one can consider the following: (1) reduced access to ions such as calcium (Martins et al., 2011a) needed by ancillary components of the pump, possibly those which induce conformational changes of the fusion proteins, needed to produce the peristaltic activity that promotes the movement of water and compounds through the pump (Seeger et al., 2008); (2) inhibiting access to the energy provided by the PMF [example, carbonyl cyanide *m*-chlorophenyl hydrazone (CCCP); Varga et al., 2012]; (3) inhibiting metabolic enzymes that yield hydronium ions needed for the maintenance of the PMF (Amaral et al., 2011a); (4) competing with antibiotics or other substrates for access to the EP (example, PAβN; Martins et al., 2009b); (5) simply by non-specific blocking (coating) of the bacterial envelope (Amaral et al., 2000).

Phenothiazines are heterotricyclic compounds, which have given rise to a large amount of medicinal compounds developed during the twentieth century. Due to their planar structure, they readily intercalate between nucleic bases of DNA inhibiting replication of the cell. They also have varying degrees of affinity for outer cell membranes of Gram-negative bacteria (Amaral et al., 2000) and plasma membranes of eukaryotes. The first neuroleptic phenothiazine chlorpromazine (CPZ) was introduced by Rhone-Poulenc in the late 1950s for therapy of psychosis and because it was immediately used world-wide, many of the serious side effects produced by CPZ were studied. As a consequence, a variety of medical research avenues opened up. Among these avenues was the study of the effects of CPZ on mycobacteria (Amaral et al., 1996; Kristiansen and Amaral, 1997; Amaral and Kristiansen, 2000) and other pathogenic bacteria (Amaral and Lorian, 1991; Amaral et al., 1992). Because CPZ was shown to increase the activity of some antibiotics to which the bacterium was resistant (Amaral et al., 1992), this agent and other phenothiazines that produce much milder side effects than CPZ, were studied for their effects on EPs of MDR bacteria (Kristiansen et al., 2003, 2006).

The phenothiazine TZ has its origins in CPZ. It is superior to CPZ in that it affords good therapeutic control of the psychotic patient without exposing the patient to the plethora of serious negative side effects. As was the case with CPZ, global use of TZ yielded a large number of studies, which eventually led to its potential use as an adjunct to antibiotic therapy for extensively drug resistant tuberculosis (XDR-TB; Amaral et al., 2010a, 2012; Abbate et al., 2012; Amaral and Viveiros, 2012). Because the mechanism of action in part involves the ability of TZ to inhibit the EPs of *Mycobacterium tuberculosis* (Amaral et al., 2004; Rodrigues et al., 2009, 2012; Dutta et al., 2010, 2011; Machado et al., 2012), focus on the EPs of the Gram-negative *E. coli*, as a model for study, took place (Amaral et al., 1992, 2011a; Viveiros et al., 2005, 2007; Martins et al., 2009a, 2012, 2013a) and a variety of new methods were introduced for the identification of MDR strains that over-expressed their EP system (Couto et al., 2008; Viveiros et al., 2008, 2010; Martins et al., 2011b, 2013b; Martins and Amaral, 2012). These methods were also useful for the evaluation of agents for activity against the EP of bacteria (Couto et al., 2008; Viveiros et al., 2008, 2010; Martins et al., 2011b, 2013b; Martins and Amaral, 2012).

## THE ETHIDIUM BROMIDE-AGAR METHOD

The simplest method for the demonstration of an over-expressed EP system of pathogenic bacteria involves the over-night cultures of the MDR clinical isolate and reference strains that represent the wild-type and their counterparts that over-express EP system(s). A dipped swab or loopful of these cultures is swabbed or streaked evenly on the surface of a series of plates that contain varying concentrations of ethidium bromide (EB) in a suitable agar of pH 7. The agar on the plate can be divided into sectors if more than one strain is to be simultaneously studied for EP activity. The plates are then incubated over-night at 37°C and examined under UV light for evidence of pink fluorescence. The lowest concentration of EB that is associated with the presence of pink fluorescence is recorded. While the wild-type counterpart reference strain begins to show evidence of fluorescence at a given low concentration of EB, the MDR clinical strain whose MDR phenotype is due primarily or partially to an over-expressed EP will begin to exhibit fluorescence at a much higher concentration of EB. If done properly, given to its simplicity, the method is almost always successful. Examples of this method with *S. aureus* strain ATCC25923 and *S. aureus* methicillin-resistant (MRSA) strains COL (Kornblum et al., 1986) and HPV107 (Sanches et al., 1995; Costa et al., 2010) are described in detailed by Martins et al. (2010).

The method can be used for a simultaneous clear-cut demonstration of two strains that differ with respect to the presence/absence of an EP. As an example, a swab of a mixture of *E. coli* K12 AG100 (whose main efflux pump acrAB is intact) and *E. coli* AG100A (whose main efflux pump *acrAB* is deleted; Viveiros et al., 2005, 2007) on agar containing increasing concentrations of EB, affords the distinction between the two strains with respect to differential efflux activity. Whereas the strain AG100A fluoresces at the EB concentration of 0.6 mg/L, the AG100 strain begins to show evidence of fluorescence at much higher concentrations of EB. The method has also been used for the demonstration of the loss of plasmids that carry genes coding for given EPs (Costa et al., 2010).

The EB agar method can be used for simultaneous determination of the activity of EP of as many as 12 strains each one of which is streaked with a swab dipped into over-night culture that had been diluted to 0.5 of the McFarland scale (Martins et al., 2011b, 2013b). Alternatively, a culture that is monitored at 600 nm until its OD reaches 0.6 can be used. When the culture reaches an OD of 0.6, its contents are washed three times to remove any trace of medium and suspended in phosphate buffered saline (PBS, pH 7), its OD adjusted to 0.6 and then swabbed or streaked onto the agar. It can be used to assess the response of strains to agents that are known to inhibit EP activity (Martins et al., 2011b) or are being studied for possible inhibitory activity (Martins et al., 2010). For those laboratories that do not wish to use EB for a variety of reasons, an alternate method has been developed which employs acridine orange (AO) as the tracer fluorescent agent (Martins and Amaral, 2012). Either EB or AO in plain agar can be used to characterize the EP system with respect to energy and needs for distinct ions. In other words, the agar is dissolved in defined minimal media that satisfies the physiological needs of the study and thereby can replace the general agar/whole media approach. Similarly the EB or AO containing agar readily affords the study of the effects of pH/temperature on efflux activity.

## THE AUTOMATED EB METHOD

The EB agar method, although useful for a variety of studies of EPs, does not provide information on a real-time basis. A more sophisticated method has been developed which utilizes the Corbett Research 3000^TM^ thermocycler for the evaluation of efflux and assessment of agents that affect efflux on a real-time basis under defined physiological conditions such as time (kinetics), temperature, pH, ions, EPIs, etc. (Paixão et al., 2009; Viveiros et al., 2010). The method uses EB although AO can be used as well (Martins and Amaral, 2012) for tracing efflux events in defined medium such as saline, pH, etc. The method utilizes an inoculum from a culture that has reached an OD of 0.6 at 600 nm and which has been washed three times to remove any trace of the medium, re-suspended in PBS and its OD adjusted to 0.6 at 600 nm. Aliquots of 50 μL are transferred to microtubes (volume of 200 μL) separately containing 50 μL of control PBS with/without a source of energy (glucose or other energy providing compound) and given pH and to separate microtubes containing 50 μL of the experimental PBS (pH, ions, energy, etc.). The tubes are placed into the thermocycler that has been programed for temperature, interval of reading, length of evaluation (minutes). The instrument exposes each tube during its centrifugation at low speed to an excitation wavelength (535 nm) and evaluates the emission from each tube at 585 nm. The results are provided on a real-time basis and available at any time during the assay for easy comparison of accumulation differences. From experiment to experiment, the variation is less than 10%. The complete method is described in detail (Paixão et al., 2009; Viveiros et al., 2010). The method has been used for the evaluation of compounds for activity against the EP system of wild-type, EP deleted, and over-expressed EPs (Cerca et al., 2011; Machado et al., 2011; Takács et al., 2011; Dymek et al., 2012). In addition, the method has also been useful for determining competition between the EP substrates PAβN and EB resulting in the determination of the Michaelis constant for PAβN (Martins et al., 2009b) and for competition between the antibiotic EP substrate TET and EB (Cerca et al., 2011). Of possible interest to readers who are involved in the study of EPs of eukaryotes, the EB automated method has been adapted for the assessment of the ABCB1 transporter of MDR cancer cells (Spengler et al., 2009b) and agents that affect the activity of this pump (Spengler et al., 2009a, 2010).

The method has been significantly useful to assess the response of *Salmonella* Enteritidis exposed to phenothiazines and the activity of its genes that regulate EPs and code for the components of the RND AcrAB-TolC pump (Spengler et al., 2012). Prior studies have shown that the phenothiazine CPZ inhibits the replication of the organism during the first 8 h of exposure after which the organism develops resistance to a concentration of CPZ as high as 100 mg/L (Amaral et al., 2000). Consequently, if one observes only the final results of growth after 24 h of exposure, one would miss the early responses of the organism, which are clearly those of susceptibility to the agent. Since CPZ is an EPI which at high concentrations inhibits the replication of the bacterium, we had expected that during a 24-h culture the inhibitory effects of the phenothiazine would be noted throughout the culture period. Moreover, our attempts to induce resistance of *E. coli* to either CPZ or TZ via serial exposure to increasing concentrations of these agents indicated that unlike the response of this organism to an antibiotic such as TET (Viveiros et al., 2007), no increased resistance took place (unpublished observations). This difference in response to an EPI was considered to perhaps be due to the major difference between *Salmonella* sp. and *E. coli*; the former which has the global EP regulator gene *ramA* which is absent in the latter Gram-negative species (Pagès and Amaral, 2009). During the first 8 h of exposure to the phenothiazine TZ, the growth of *Salmonella* Enteritidis is inhibited whereas progressive increased activity of the genes that regulate and code for the AcrB efflux pump takes place such that by the end of 8 h the organism becomes increasingly resistant to TZ (**Table 2**). These results also support the notion that in order to define the response of a bacterium to an agent, the bacterial culture containing the agent must be monitored during the exposure period for growth and for the expression of key genes that may increase/reduce resistance.

**Table 2:** Activities of genes during transient inhibition of growth from exposure to 100 mg/L thioridazine (TZ) on *Salmonella enterica *serovar Enteritidis 104.

	Relative expression quantification (2^-ΔΔCt^				
Genes	0.5 h	1 h	4 h	8 h	16 h
*soxS*	0.9	12.1	1.9	0.0	0.1
*rob*	2.3	1.4	2.0	0.5	2.0
*ramA*	3.2	5.7	45.3	14.9	1.5
*marA*	0.4	0.7	10.6	0.9	0.1
*acrB*	6.1	–	104.0	315.2	8.0
*pmrA*	1.6	2.0	5.3	9.8	0.9
*pmrB*	0.5	2.1	13.0	3.7	0.7

*During the first period of no growth (first 8 h), the genes that regulate and code for the AcrB transporter are sequentially activated; first *soxS*, then followed later by *ramA, marA*, and *pmrB*, and then by 8 h of culture *ramA* decreases its activity, *marA* returns to baseline activity, *acrB* is maximally increased in activity and *pmrA* is now active. By the end of the culture period (16 h), only *acrB* is over-expressed.
*

## THE RELATIONSHIP OF THE EFFLUX PUMP TO TWO-COMPONENT RESISTANCE REGULONS

Subsequent to the phagocytosis of the Gram-negative *Salmonella* sp. by the neutrophil, the acidic pH of the phagolysosome promotes the activation of the organism’s two-component resistance regulon PmrA/PmrB (Gunn, 2008). pH activates the sensor PmrB to undergo self-phosphorylation after which it acts as a kinase and transfers the phosphate to PmrA. Activation of the *pmrA* gene then activates a cascade of genes, which promote synthesis of Lipid A and its introduction into the nascent LPS of the outer cell wall of the organism. Perhaps, as many as 100 other genes are also activated (Gunn, 2008). The increase of LPS renders the organism resistant to the hydrolytic enzymes of the phagolysosome. Activation of the PmrA/PmrB regulon promotes the activation of the *pmrD* gene whose product activates the global EP regulator gene *ramA* which in turn results in the activation of the transporter coding gene *acrB*. The activation of the PmrA/PmrB regulon therefore renders the intracellular trapped *Salmonella* sp. practically resistant to most agents. It is for this reason that a patient who has recently undergone a resection of the colon and has ingested food contaminated with *Salmonella* sp., is very difficult to successfully treat.

Mice can be protected from an infection by the highly virulent *Salmonella* Typhimurium 74 by pre-treatment with clinically relevant doses of the phenothiazine TZ although the MIC of the phenothiazine against the organism is many-fold greater than the level of the drug in its blood (Dasgupta et al., 2010). Because a heavy inoculum is introduced directly into the neutrophil-rich peritoneum, it is opined that the protection from infection results from the highly concentrated level of TZ within the lysosomes of the neutrophil (Daniel and Wójcikowski, 1999a,b) that eventually would fuse with the phagosome that contains the phagocytosed organism. The high concentration of TZ within the phagolysosome may exceed the *in vitro* MIC of TZ and because TZ can readily penetrate the cell envelope of the bacterium (Motohashi et al., 2003), it can easily reach sensitive TZ targets such as DNA where it readily intercalates between nucleic bases of DNA (Crémieux et al., 1995). The fact that TZ also enhances killing of intracellular bacteria (Crémieux et al., 1995; Ordway et al., 2003; Martins et al., 2004, 2009c; Amaral et al., 2007), suggests that the protection by TZ from a virulent infection takes place via many mechanisms of action.

## THE RELATIONSHIP OF THE QUORUM SENSING SYSTEM AND SECRETION OF BIOFILM TO THE EFFLUX PUMP

### QUORUM SENSING AND ITS ROLE IN INFECTION

Communication between bacteria of the same species and between species, also termed quorum sensing (QS), contributes to their survival (Varga et al., 2012). It involves the secretion of signaling molecules that induces specific responses from the targeted bacteria, for example: (1) reduction of population growth of a species and hence, reducing the possibility of exceeding the nutritional support of the environment; (2) inhibition of replication of, or even killing (biocides) of competing species; (3) promotion of swarming that recruits members of the same species to migrate to a specific location (Szabó et al., 2010; Amaral and Molnar, 2012; Varga et al., 2012); (4) secretion of materials that will protect the bacterium from external danger. In the latter case, these materials can form a matrix of polysaccharides that involves the bacteria (from the same species or even from different species), termed biofilm. Within the biofilm, channels are formed and used for further communication between the bacteria. Biofilms are produced in nature at sites such as surfaces of rocks, which maintain the bacterial population *in situ*, or at sites of the human colonized by infecting bacteria, such as the surface of prosthetic devices after their placement, which can lead to development of an infection. The presence of biofilm renders therapy of the infection problematic. The QS responses of the infecting bacterium are obviously important and consequently, the search for such compounds that are able to inhibit the QS system and biofilm formation has been in effect for the past two decades.

There is a relationship between EPs, QS, and biofilm secretion, which has come to the forefront only recently (Varga et al., 2012). Control of this relationship is critical for successful therapy of MDR bacterial infections which have become rather commonplace.

Inhibitors of bacterial QS systems must be distinguished from their activity on the producer of the signal, the responder to a QS signal or both. QS inhibitors (QSIs) are compounds that specifically block QS systems without affecting bacterial growth. The QS system of bacteria that has received the greatest attention is the acyl homoserine lactone (AHL) system which produces and secretes AHL. AHL acts as a communication molecule which regulates the behavior of the members of the bacterial population (Amaral and Molnar, 2012). Concerted behavior such as swarming of bacteria, production of surfactant, which facilitates bacterial movement on surfaces, production and secretion of virulence factors and biofilms, are examples among a growing list of concerted behaviors (Szabó et al., 2010). Obviously, the regulation of bacterial behavior and population density by AHLs suggests activity at the genetic level. The AHLs density dependent regulatory systems rely on two proteins, an AHL synthase, most commonly a member of the LuxI family of proteins, and an AHL receptor protein belonging to the LuxR family of transcriptional regulators (Ordway et al., 2003; Varga et al., 2012). Low population density cells produce a basal level of AHL that is dependent on an AHL synthase. With increase of population density, AHL accumulates in the medium and when it reaches a critical threshold concentration, the AHL molecule binds to its cognate receptor (Ordway et al., 2003; Martins et al., 2004). The binding of AHL to its receptor promotes induction or repression of AHL-regulated genes. The genes that are regulated are responsible for a large number of functions such as bioluminescence, plasmid conjugal transfer, biofilm formation, motility, antibiotic biosynthesis, production of virulence factors, among others.

The inhibition of the QS system of pathogenic bacteria is a goal of drug discovery. Perhaps the best known inhibitors of the AHL QS system are homologs of AHL, and are produced by other bacterial species. *N*-butanoyl-L-homoserine lactone (C4-HSL) and *N*-hexanoyl-L-homoserine lactone (HHL) are produced in cultures of *Serratia liquefaciens* and serve as autoinducers of swarming. However, analogs of C4-HSL such as *N*-(3-oxododecanoyl)-L-homoserine lactone (3-oxo-C(12)-HSL) produced by *Pseudomonas aeruginosa* do not affect cell growth of its own population nor that of *Proteus mirabilis, E. coli, Alcaligenes faecalis*, or *Stenotrophomonas maltophilia*, but inhibit the growth of *Legionella pneumophila* as well as the formation of its biofilm (Amaral and Molnar, 2012). Among compounds that inhibit production and secretion of AHL are sulfur-containing AHL-analogs such as *N*-(heptylsulfanylacetyl)-L-homoserine lactone (HepS-AHL) which reduces production of protease by *Aeromonas salmonicida*, rendering the bacterium less virulent (Szabó et al., 2010).

Because both QS and biofilm formation involves secretion of compounds, efflux systems of the cells should be involved in this process. In fact, phenothiazines, which inhibit many energy dependent systems of bacteria including some of its EPs, also inhibit QS signaling (Amaral and Molnar, 2012; Varga et al., 2012). Consequently, the inhibition of an EP should result in the inhibition of the QS component responsible for biofilm formation.

During the past two decades drug development and discovery have focused on plants as sources of bioactive compounds. In particular, since the discovery of berberine, a powerful inhibitor of bacterial EPs, plants have become sources of inhibitors of EPs and, consequently, of QS systems. For example, essential oils yield a large number of compounds that inhibit the QS system of responding bacteria; some of them have promising inhibitory properties for the short chain AHL QS system in *E. coli* containing the biosensor plasmid pJBA132. Citral is the only essential oil that presented some activity for the long chain AHL QS system in *Pseudomonas putida* containing the plasmid pRK-C12. Because some essential oils have also been shown to inhibit the EP of antibiotic resistant Gram-negative bacteria, the relationship between EPs and the QS of bacteria seems, once more, is well established. EPs of Gram-negative bacteria that bestow MDR are mostly dependent upon the PMF for activity. Studies reveal that some compounds that inhibit the EP of bacteria supposedly by the inhibition of the PMF energy source are highly active as inhibitors of the QS response (Varga et al., 2012). These latter studies serve to support the intimate connection between EPs and the QS system of bacteria. Consequently, compounds that affect both are good candidates against the secretion of biofilm matrix which is dependent upon the QS system and the EP for secretion of the biofilm.

## CONCLUDING REMARKS

The EPs of bacteria, as demonstrated by this review, do more than simply recognize external and internal noxious agents for extrusion to the milieu in which the organisms live. They are connected to a variety of resistance mechanisms that together serve to assist the bacterium to survive in environments that are toxic. The mechanisms such as QS responses, biofilm production and secretion, and those two component regulons are intimately tied to the activity of the EP system of the organism. Moreover, as indicated in this review, because the EP system is deemed by us to be the main path for the passage of water formed from metabolic activity, the passage of acidified water serves the purpose of reducing the pH of the internal component of the transporter, thereby allowing the release of any bound EP substrate which in turn is carried in the jet of water and extruded via the TolC channel. Concept-wise, we may consider the EP system as the excretory organ of the bacterium performing those functions of multi-cell animals such those of the digestive and urinary tract systems. Nevertheless, as discussed in this review, the targeting of the EP system may also obviate other resistance mechanisms and hence, this approach appears to be worthwhile.

## Conflict of Interest Statement

The authors declare that the research was conducted in the absence of any commercial or financial relationships that could be construed as a potential conflict of interest.
